# Effectiveness of Violence Prevention Interventions: Umbrella Review of Research in the General Population

**DOI:** 10.1177/15248380231195880

**Published:** 2023-08-31

**Authors:** Seena Fazel, Matthias Burghart, Achim Wolf, Daniel Whiting, Rongqin Yu

**Affiliations:** 1 University of Oxford, UK; 2 University of Konstanz, Germany; 3 University of Nottingham, UK

**Keywords:** violence, prevention, interventions, umbrella reviews, meta-analysis, treatment effectiveness

## Abstract

To address the societal harms of violence, many violence prevention interventions have been developed, tested, and implemented in the general population. These have been reported in systematic reviews and meta-analyses, which have typically focused on one type of intervention or outcome. We aimed to provide a comprehensive overview of the current evidence regarding the effectiveness of different psychosocial interventions in reducing all forms of violence toward others. We have conducted an umbrella review of previous meta-analyses using standard approaches and converted findings on effectiveness into odds ratios. We tested for the underlying quality of the meta-analytic evidence by examining heterogeneity, excess statistical significance, prediction intervals, and small study effects. We identified 16 meta-analyses, including nine investigating psychosocial interventions, and five legislative and policy changes. Most meta-analyses reported positive effects of tested interventions. The strongest effects were found for sports-based initiatives, and the weakest for general population programs aimed at early childhood, youth development, and reducing sexual assault perpetration by men. Legislative changes had varying effectiveness. We conclude that simple, scalable, and cost-efficient programs, such as sport-based initiatives, have the clearest empirical support as population-based approaches to violence prevention.

Violence against others is a public safety and health problem globally (World Health Organization, 2014, 2022). It is characterized as any intentional use of physical force or power, threatened or actual toward another person that either results in or has a high likelihood of causing injury, death, or psychological harm. It has severe impacts on the physical and psychological morbidity of victims at the population level. The economic burden is considerable with estimates that interpersonal violence costs globally $15 trillion annually or 12% of the worldwide gross domestic product (Iqbal et al., 2021). On an individual level, research has consistently shown that both violence perpetration and victimization are associated with negative behavioral and health-related outcomes. In young people, these include poorer educational outcomes and an increased risk of premature mortality (Fry et al., 2018; Smiley et al., 2021). Across all ages, increased risks of psychiatric symptoms and diagnoses, suicidal behaviors, and further violence have been reported (de Ruiter et al., 2022; Hailes et al., 2019; MacIsaac et al., 2017; Smith et al., 2020; Wright et al., 2019). It is also linked to psychological effects on families and carers of victims, healthcare workers, and community-related harms.

To address these harms, many violence prevention interventions have been developed. These include universal ones aimed at the general population, and targeted ones for those at increased risk for violent behavior (e.g., individuals who misuse substances). In addition, indicated interventions, directed at individuals who have perpetrated violence before (e.g., convicted persons), have been tested. Regardless of the level of intervention, violence prevention programs need to be supported by evidence of their efficacy (World Health Organization, 2022). However, most violence intervention programs have not been properly tested (Kovalenko et al., 2022; Nation et al., 2003), some of which may be harmful as they might increase the risk of violence or other adverse outcomes. Those with no effect on individual outcomes are also harmful in wasting limited resources. The proliferation of new interventions can mean that policymakers are unable to keep up and appraise new evidence (Kovalenko et al., 2022; Matjasko et al., 2012). Although there has been an increase in systematic reviews that aim to summarize the evidence, they are typically limited in terms of selecting one intervention or outcome to examine (e.g., Eggers del Campo & Steinert, 2022). They are also limited by their methodological quality, which varies widely and results in conflicting findings, which further complicates interpretation of the evidence.

One comprehensive summary of “what works” in violence prevention is a meta-review that is a decade old (Matjasko et al., 2012), which examined the effectiveness of youth violence prevention programs. Its findings suggested that interventions based on cognitive-behavioral therapy, parental training, peer mediation, or certain school-based approaches were most effective at reducing violence among adolescents. Interventions based solely on deterrence (e.g., “Scared Straight,” which organizes visits to correctional facilities by juveniles who have offended or children at risk of doing so), on the other hand, were associated with increased offending risk. This meta-review is now dated and did not make a clear distinction between the different levels of intervention (i.e., universal, targeted, and indicated). This is potentially important as previous research has shown that universal violence interventions are often less effective than targeted and indicated ones (Dodge, 2020). In addition, the previous meta-review lacked a quality assessment of included reviews and quantitative synthesis, and was limited to youth violence, rather than including interventions for individuals of all ages. Therefore, it is important to address this gap in the literature and clarify the effectiveness of universal interventions, due to its potential impact at a population level and in reducing societal costs of violence and antisocial behaviors.

## The Present Umbrella Review

To address limitations in the previous literature, we conducted an umbrella review of the evidence on the effectiveness of universal violence prevention interventions for all age groups. An umbrella review is a synthesis of existing systematic reviews and allows the findings of reviews to be compared and contrasted, allowing for a more comprehensive analysis of the current evidence than any individual review on a specific topic (Aromataris et al., 2015). In addition, umbrella reviews can summarize systematic reviews using a structured quality assessment and are increasingly used to obtain a clearer overview of a field where there is a large research literature and where individual systematic reviews may have reported conflicting findings. A previous umbrella review examined targeted interventions in mental health populations (Wolf et al., 2017). Thus, the objective of the current study is to provide a comprehensive overview of the effectiveness of psychosocial interventions for violence in the general population.

## Methods

### Search Strategy and Eligibility Criteria

We conducted a comprehensive systematic search strategy following the Preferred Reporting Items for Systematic Reviews and Meta-Analyses (PRISMA) guidelines (Moher et al., 2009). In all, 10 databases were searched from inception to April 2022: Cochrane Database of Systematic Reviews, DARE, Epistemonikos, JBI Database of Systematic Reviews and Implementation Reports, Medline, PsycINFO, PROSPERO, PubMed, Scopus, and Web of Science. In each database, the same combination of the following search terms was used to search the title, abstract, and keywords of an article: (((prevent* OR *risk management* OR *risk reduction* OR deter*) AND (violen* OR homicid* OR assault* OR *rape* OR robber* OR bully*)) OR (recidiv* OR reoffend* OR repeat offend*)) AND (*systematic review* OR *meta-analysis*). In addition, reference lists of relevant reviews and Google Scholar were hand-searched.

A review was eligible for inclusion if it was a meta-analysis that examined the effectiveness of a universal violence prevention intervention (i.e., not including reviews that focused on interventions for high-risk populations or individuals with a history of violent behavior) and reported outcome data for interpersonal violence perpetration. Violence was defined based on an adapted WHO definition for individuals (intentional use of physical force or power, threatened or actual toward another person that either results in or has a high likelihood of resulting in injury, death, or psychological harm) and did not include self-directed violence (i.e., self-harm or suicide), violence victimization, or attitudes towards violence. Both published and unpublished reviews in any language were considered. Primary studies and reviews with methodologies other than a meta-analysis (e.g., systematic reviews, meta-reviews) were excluded as we intended to provide quantitative comparisons and assess quality.

### Data Extraction

Data extraction followed a two-stage process. First, titles and abstracts of all articles retrieved from the systematic search were screened and excluded from further consideration if inclusion criteria were not met. Then studies were read by two independent reviewers (MB and AW) and, if eligible for inclusion, entered into a standardized data extraction form. Extracted data included relevant information on the population, intervention, outcome, setting, number of included studies and participants as well as statistical information including effect size, confidence and prediction interval, level of heterogeneity, and the meta-analytical model used. Where data were missing, corresponding authors were contacted by email. Any conflicts that arose during the extraction process were resolved in consultation with SF.

### Data Analysis

Because the reported effect sizes varied across reviews, these were converted to a common metric, namely odds ratios (ORs). Statistical approximations were used for all conversions. In instances where no formula exists to directly calculate an OR, the effect size was first converted to Cohen’s *d* (Borenstein et al., 2011). In addition, the final effect sizes were transformed such that OR values >1 indicate that the intervention resulted in violence reduction, whereas OR values <1 indicate an unfavorable effect.

When reviews reported pooled effect sizes for separate interventions, each intervention was included in the umbrella review as a distinct and independent effect (Higgins et al., 2019). If, however, multiple pooled effect sizes were calculated as part of a moderator analysis, only the one with the highest quality rating (see below) was considered for all subsequent analyses (e.g., follow-up measures were preferred over measures taken immediately after the intervention). In addition, as several eligible articles examined the effectiveness of universal and targeted violence prevention interventions simultaneously, it was necessary to disentangle their findings (Aromataris et al., 2015). That is, the statistical analysis of the included meta-analysis was repeated by including only those primary studies that focused exclusively on universal prevention strategies. In the absence of the data required to rerun the analyses (e.g., missing standard errors), these were requested from the corresponding author. When authors did not respond or could not provide the information, it was approximated from forest plots using the WebPlotDigitizer R-package (Rohatgi, 2022). Finally, to prevent overlap between reviews, only the largest meta-analyses of those with overlapping primary studies were included in our main analyses.

### Quality of Evidence

The overall quality of each included meta-analysis was assessed using six different approaches proposed in previous umbrella reviews (Bellou et al., 2017; Fazel et al., 2018). First, the Assessing the Methodological Quality of Systematic Reviews (AMSTAR; Shea et al., 2007) was scored. The AMSTAR consists of 11 items that are summed to produce a final score indicating low (0–3 points), medium (4–7 points), or high (8–11 points) methodological quality. Second, the ratio between the pooled overall effect size of a meta-analysis and the effect size of its largest included study was calculated as a measure of statistical excess bias (Kavvoura et al., 2008). Since the largest included study is considered the most accurate (Lipsey & Wilson, 2001), a ratio >1 is a strong indication of the presence of excess statistical significance (Kavvoura et al., 2008). Third, the between-study heterogeneity within each review was quantified using the *I*^2^ statistic (Higgins et al., 2019). *I*^2^ quantifies the proportion of variability across studies that is not due to chance. Values >50% were considered large (Ioannidis et al., 2007; Solmi et al., 2018). Fourth, the 95% prediction interval of a review’s overall pooled effect size was inspected (IntHout et al., 2016). Prediction intervals that include the null effect (i.e., OR = 1) indicate potentially nonsignificant findings in a new population (Higgins et al., 2019; Riley et al., 2011). Fifth, Egger’s regression asymmetry test was used to assess small-study effects (Egger et al., 1997). Significant results in this test were considered evidence of publication bias (Sterne et al., 2011). Sixth, reviews with more than 1,000 participants were rated as being of higher quality than reviews with fewer participants given the greater statistical power of larger meta-analyses (Borenstein et al., 2011). Finally, to summarize these distinct quality assessments, they were aggregated into an overall quality score, ranging from 0 (low quality) to 6 (high quality). Missing data on quality criteria were scored as 0.

## Results

### Study Characteristics

The systematic literature search yielded a total of 5,378 articles. After screening titles and abstracts, the full texts of 116 papers were reviewed for eligibility, resulting in 30 meta-analyses. When overlap between these reviews was accounted for, 16 meta-analyses with 22 pooled effect sizes remained in our main analyses. Results of all other eligible reviews are reported in Supplemental Table 1.

Included meta-analyses were published between 2010 and 2022. The number of included participants ranged from 400 to 35,000, with a median of 5,546. The majority of reviews (*k* = 9) synthesized evidence for psychosocial interventions, most of which consisted of parent and teacher training on antibullying strategies and child skills training (e.g., social and emotional learning, recognition of dating violence). Five articles examined legislative and policy changes such as increasing alcohol taxes, expanding closed-circuit television (CCTV) surveillance, vacant lot remediation (i.e., greening, mowing, gardening of unused land), and implementing conservative gun laws. Violence prevention interventions based on physical activity (e.g., martial arts) were examined in two reviews (Harwood et al., 2017; Spruit et al., 2016). Other strategies include female economic empowerment and male-targeted sexual assault prevention programs. Overall, outcomes varied considerably across reviews, including aggression, cyberbullying, bullying, gun violence, disruptive behavior, violent crime, and sexual violence (see Supplemental Table 2a for details). Most interventions primarily focused on addressing bullying behaviors, including cyber and physical bullying, conduct problems, and other antisocial behaviors such as dating violence, particularly among adolescents and young adults. However, in adult groups, outcomes tended to be more severe, including violent crime, sexual and intimate partner violence, and gun violence. Designs used to test interventions varied considerably (Supplemental Table 2b), although most were quasi-experimental studies (with a control group) or investigations that involved outcome assessment conducted before and after an intervention. Only one meta-analysis was based solely on randomized controlled trials (RCTs; Eggers del Campo & Steinert, 2022).

### Main Findings

**Figure 1.**
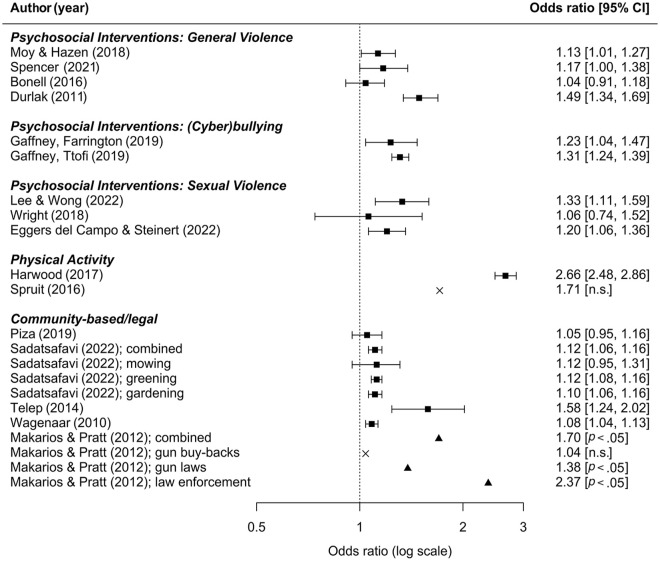
Effectiveness of universal violence prevention interventions from previous meta-analyses. *Note*. ■ = confidence interval was given; ▲ = no confidence interval given, but the *p* value reported as <.05; × = no confidence interval given, but results reported as statistically nonsignificant (n.s.).

ORs ranged from 1.04 to 2.66 with a median OR of 1.19. All 16 meta-analyses reported positive effects of intervention (i.e., OR > 1). That is, interventions were associated with a lower risk of the targeted prevention outcomes such as violence and other antisocial behaviors. As tested interventions were heterogeneous, results were also assessed separately for different types of intervention programs (Tables 1 and 2; Figure 1). The largest effect size was found for interventions based on physical activity, particularly one focused on martial arts training. Some community-based changes were associated with strong effects, such as law enforcement for gun control, but there was variation between various policy and legal initiatives. Some community-based interventions had no effects, such as gun buy-back programs and CCTV surveillance. Legislative changes around alcohol price and availability had small effect sizes. Psychosocial interventions against bullying and cyberbullying, such as the KiVa program, which focuses on bystanders, teaching children to recognize and respond when they see bullying, reported positive findings but with small effect sizes. Psychosocial programs targeting sexual and general violence through youth development interventions (delivered online), male-specific sexual assault programs, and female economic empowerment had broadly similar findings but with more variation and wider confidence intervals.

**Table 1.** Effect Sizes of all Meta-Analyses Assessing the Effectiveness of Universal Violence Prevention Interventions (Ranked by Quality Score).

Study	*k*	*n*	Quality Score	OR [95% CI]
Psychosocial interventions: general violence				
Moy and Hazen (2018)	16	7,890	4/6	1.13 [1.01, 1.27]
Spencer et al. (2021)^a^	4	1,430	4/6	1.17 [1.00, 1.38]
Bonell et al. (2016)	3	3,201	3/3	1.04 [0.91, 1.18]
Durlak et al. (2011)	112	—	2/2	1.49 [1.34, 1.69]
Psychosocial interventions: (cyber) bullying				
Gaffney, Farrington, et al. (2019)	18	34,826	3/6	1.23 [1.04, 1.47]
Gaffney, Ttofi, et al. (2019)	81	—	2/5	1.31 [1.24, 1.39]
Psychosocial interventions: sexual violence				
Lee and Wong (2022)	17	18,946	3/6	1.33 [1.11, 1.59]
Wright et al. (2018)	5	406	2/4	1.06 [0.74, 1.52]
Eggers del Campo and Steinert (2022)^b^	14	24,079	1/4	1.20 [1.06, 1.36]
Physical activity: externalizing behaviours				
Harwood et al. (2017)	8	459	2/4	2.66 [2.48, 2.86]
Spruit et al. (2016)	6	—	0/1	1.71 [n.s.]
Community based/legal: general/gun/sexual violence				
Piza et al. (2019)	29	—	3/5	1.05 [0.95, 1.16]
Sadatsafavi et al. (2022); combined	10	76,818^c^	3/6	1.12 [1.06, 1.16]
Sadatsafavi et al. (2022); mowing	3	21,526^c^	3/6	1.12 [0.95, 1.31]
Sadatsafavi et al. (2022); greening	5	54,044^c^	3/6	1.12 [1.08, 1.16]
Sadatsafavi et al. (2022); gardening	2	1,248^c^	3/6	1.10 [1.06, 1.16]
Telep et al. (2014)	4	—	3/5	1.58 [1.24, 2.02]
Wagenaar et al. (2010)	10	—	1/6	1.08 [1.04, 1.13]
Makarios and Pratt (2012); combined	29	—	0/1	1.70 [*p* < .05]
Makarios and Pratt (2012); gun buy-backs	—	—	0/1	1.04 [n.s.]
Makarios and Pratt (2012); gun laws	—	—	0/1	1.38 [*p* < .05]
Makarios and Pratt (2012); law enforcement	—	—	0/1	2.37 [*p* < .05]

*Note.* Quality score ranges from 0 (low quality) to 6 (high quality), and reports: no. positive quality items/no. quality items reported. “Mowing” interventions: trash/debris removal, mowing vegetation regularly. “Greening” interventions: trash/debris removal, grading land, planting new grass/trees, installing fences, maintaining lots. “Gardening” interventions: grading soil, planting turfgrass, mowing, and various landscaping. *k* = number of studies included in a meta-analysis; *n* = number of participants included in a meta-analysis; OR = odds ratio; CI = confidence interval; n.s. = not significant; RCTs = randomized controlled trials.

a The analyses were rerun without one clear outlier (OR > 230), which skewed the overall effect size due to the use of a random effects model. When the outlier is included in the analysis, the overall effect is 2.79 [1.15, 6.77].

b Meta-analysis based entirely on RCTs.

c Number of lots observed.

*p* < .05 = statistically significant with a confidence level smaller than .05.

**Table 2.** Critical Findings.

1	Sports-based initiatives could be effective population-based and scalable approaches to violent prevention.
2	Psychosocial interventions targeting early childhood, parents, and teachers of preschool and early years children have smaller effects than physical activity and sport-based programs aimed at adolescents and young adults.
3	Legislative and policy changes produced the most heterogeneous effects on violent outcomes.

**Figure 2.**
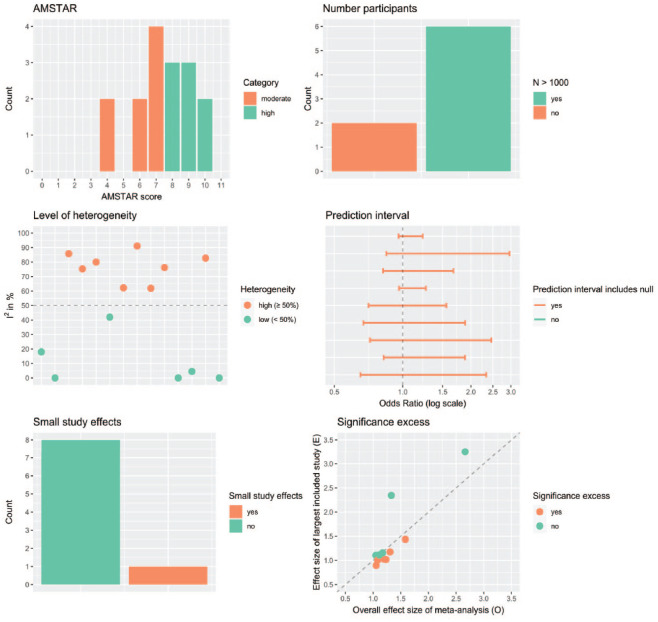
Visual representation of quality assessment. *Note*. Each panel represents the results of one of the six quality assessments performed, with orange and green generally indicating a low and high score, respectively. The sum of reviews within each panel does not always equal the total number of all included meta-analyses, since data were missing in some cases. Detailed quality assessment results for each review are shown in Supplemental Table 3.

Quality ratings indicated variable but mostly low quality in the meta-analytic literature (Figure 2; Supplemental Table 3). There were (1) wide prediction intervals, with all 16 reviews either not reporting them or including the null effect, suggesting that future studies might find no effects or opposite effects; (2) large heterogeneity across primary studies, with 16 out of 26 studies with an *I*^2^ of 50% or higher; and (3) excess statistical significance in half of the included meta-analyses. Small sample sizes (*n* < 1,000) and small study effects were found in two reviews. However, lack of information in many reviews limited the evaluation of all aspects of the quality assessment.

## Discussion

In this umbrella review, we summarized the findings of 16 meta-analyses on the effectiveness of universal violence prevention interventions. Overall, our findings suggest a mostly small but positive impact on reducing violence, with the primary effectiveness measure, calculated from effect sizes of included meta-analyses, and reported as odds ratios (ORs) ranging from 1 to 3. All the included meta-analyses reported pooled ORs higher than 1, which was also observed for the wider set of 30 meta-analyses that included overlapping underlying reviews (Supplemental Table 1). In general, we found support for sports-based and anti-bullying interventions targeting children and youth. However, there are mixed findings regarding the effectiveness of policy change programs.

The findings of this umbrella review have important implications for practice, policy, and research (Table 3). We found certain types of universal violence prevention interventions were more promising than others. Of note, the strongest effects were for interventions based on martial arts training (Harwood et al., 2017). The quality of the underlying meta-analyses also varied widely with higher-quality reviews finding small but positive effects of school-based interventions (Moy & Hazen, 2018) and an online program for intimate partner violence (Spencer et al., 2021).

**Table 3.** Implications of the Review on Effectiveness of Universal Violence Prevention Interventions for Practice, Policy, and Research.

1	Strongest effects were for sports-based interventions that provide a simple, scalable, and potentially cost-effective violence prevention measure.
2	Psychosocial interventions have smaller effects than physical activity programs aimed at adolescents and young adults.
3	Anti-bullying initiatives, which were found to have a small but consistent effect on a high-prevalence behavior, could be considered as part of any broad violence prevention strategy.
4	Simple scalable interventions, such as sports clubs, should be a primary focus for policy and research.
5	Future research should examine key shared ingredients and mechanisms that are associated with effective interventions.

We found evidence in support of sports-based interventions for reducing externalizing problems in children and youths. The review on martial arts training made a distinction between traditional and modern martial arts practices and included only the former (Harwood et al., 2017). Traditional styles (e.g., Aikido) describe internally focused techniques that emphasize self-reflection, ancient philosophies, and breathing techniques, while modern martial art forms lack these components (e.g., boxing; Hernandez & Anderson, 2015). This distinction may suggest that it is not the physical engagement in the training itself that mediates the positive effects of the intervention but rather the additional elements of traditional martial arts practices (Harwood et al., 2017). The second meta-analysis on physical activity interventions (Spruit et al., 2016) investigated sports programs in general but yielded a smaller and nonsignificant pooled effect size. This difference could be secondary to the absence of the cognitive and self-reflective components in the physical interventions studied. However, overall, such approaches provide a simple, scalable, and potentially cost-effective violence prevention measure.

Psychosocial anti-bullying programs, targeting school-aged children and youths, produced consistent evidence of effectiveness but with small effect sizes. The high base rate of bullying, estimated to be 35% in adolescents (Modecki et al., 2014), is an important context to these findings. Anti-bullying initiatives are therefore more likely to have a detectable impact than those that target less prevalent violent outcomes (Beelmann & Lösel, 2021). Another possible reason for their effectiveness is that bullying is a relatively low-severity form of violence, which is less likely to occur within a pattern of entrenched antisocial behaviors than more serious violence, and thus may be more responsive to treatment (Moffitt, 2018).

Legislative and policy changes, aiming at reducing severe forms of crime, such as general and gun violence in the general population, produced the most heterogeneous results in this umbrella review. A number of factors may explain this. First, the outcome measure for these types of studies usually has a high outcome threshold, such as criminal arrest or conviction. Second, specificity is a key principle for an effective prevention program (Nation et al., 2003); however, all but one meta-analysis (Makarios & Pratt, 2012) investigated legislation that aimed to reduce violence-related crime (e.g., burglary, vandalism) rather than violent offenses specifically. Third, it is possible that the distal effect of policy changes on violent outcomes may only be noticeable after several years and not be captured in the time span of research studies. The findings on the positive effects of alcohol legislation on price and availability on reducing violence, although associated with smaller effect sizes, are important from a population perspective due to the underlying high prevalence of alcohol use and misuse (Wagenaar et al., 2010).

In view of the high cost-benefit ratio of universal programs (Beelmann & Lösel, 2021; Greenberg & Abenavoli, 2017), this umbrella review suggests that some universal interventions, if implemented, require a review of their impact to justify their continuation. Where implementation is expensive or resource-intensive across criminal justice, health, and educational services, the highest quality evidence in support should be required before wholescale adoption. At the same time, many universal interventions are simple, relatively cheap, and quick to implement because they do not require a preselection of individuals (Beelmann & Lösel, 2021). Moreover, they are usually associated with additional benefits beyond violent reduction, such as increased prosocial skills (Durlak et al., 2011), less substance use (Bonell et al., 2016), or improved dating violence knowledge/attitudes (De La Rue et al., 2017).

### Limitations

Some limitations should be noted. First, the definition of violence was necessarily broad, which was consequently associated with expected high levels of heterogeneity. Direct comparisons between certain programs need to be made with caution due to different outcome thresholds and prevalence. Second, most primary studies in the included meta-analyses used short follow-up periods for the evaluation of interventions. Thus, the reported effect sizes are likely to be an overestimation of the true long-term effects. Third, most meta-analyses in this umbrella review did not provide sufficient data for the comprehensive quality assessment. Fourth, all included reviews reported overall positive effects, which suggests that publication and allegiance biases are prominent in this area.

In addition, the heterogeneity of the included reviews might be explained by sample characteristics and study settings. For example, information about background characteristics of the sample, such as gender and socioeconomic status, and settings in which interventions were implemented (community centers vs. clinics), should be improved and can be examined as potential explanations for the heterogeneity when this literature is updated. Furthermore, most of the studies were conducted in high-income countries, and it is not known whether findings can be generalized to low- and-middle income countries where resources are more limited. In many contexts, cultural adaptation will be required, and testing this should be part of any implementation process.

### Implications

The findings of this umbrella review have some direct policy implications. First, in contrast to much expert opinion (Lannen & Ziswiler, 2014), psychosocial interventions that have been widely implemented in high-income countries, and predominantly target early childhood, parents, and teachers of preschool and early years children, have smaller effects than physical activity programs aimed at adolescents and young adults. Therefore, such interventions, such as sports clubs, with the associated relevant facilities, should be a primary focus for policy and research. Second, anti-bullying initiatives, which were found to have a small but consistent effect on a high-prevalence behavior, could be considered as part of any broad violence prevention strategy. Overall, universal programs may be best suited as a quick, resource-efficient, and large-scale prevention method. In contrast, targeted and indicated interventions, which typically have stronger effects but are more resource-intensive, could be reserved for more severe forms of violence. These interventions may be longer in duration, require specially trained staff for delivery, and engage multiple agents (e.g., health services, family, peers, and community residents).

Future work should consider what are the key ingredients and mechanisms that explain effective interventions. In addition, the lack of stronger effects for broad psychosocial interventions, such as thinking or social skills training, suggests that more focused universal prevention approaches should be evaluated, including those based on group-based interventions and addressing modifiable risk factors, including substance misuse.

## Conclusion

Universal violence prevention interventions, particularly those aimed at early childhood, have mostly small effects on violence perpetration and require more evidence in support before further implementation. Simple, scalable, and cost-efficient programs, such as sport-based initiatives, appear to have more empirical support than other population-based approaches to violence prevention.

## Supplemental Material

sj-docx-1-tva-10.1177_15248380231195880 – Supplemental material for Effectiveness of Violence Prevention Interventions: Umbrella Review of Research in the General Population Supplemental material, sj-docx-1-tva-10.1177_15248380231195880 for Effectiveness of Violence Prevention Interventions: Umbrella Review of Research in the General Population by Seena Fazel, Matthias Burghart, Achim Wolf, Daniel Whiting and Rongqin Yu in Trauma, Violence, & Abuse
