# Supplementary material for: Effectiveness of Violence Prevention Interventions: Umbrella Review of Research in the General Population
Source: Trauma Violence Abuse. 2023 Aug 31;25(2):1709–18. doi: 10.1177/15248380231195880 (PMC10913357; doi:10.1177/15248380231195880)
Supplement: sj-docx-1-tva-10.1177_15248380231195880 – Supplemental material for Effectiveness of Violence Prevention Interventions: Umbrella Review of Research in the General Population [file sj-docx-1-tva-10.1177_15248380231195880.docx]

**Supplementary Table 1**

*Effect Sizes of all Meta-Analyses (regardless of overlap) Assessing the Effectiveness of Universal Violence Prevention Interventions (Ranked by Quality Score)*

| **Study** | ***k*** | ***n*** | **Quality Score** | **OR [95% CI]** |
| --- | --- | --- | --- | --- |
| **Psychosocial Interventions: General Violence** | | | | |
| Langford et al. (2014) | 11 | 35,449 | 4/6 | 1.25 [1.08, 1.45] |
| Moy & Hazen (2018) | 16 | 7,890 | 4/6 | 1.13 [1.01, 1.27] |
| Spencer et al. (2021)^a^ | 4 | 1,430 | 4/6 | 1.17 [1.00, 1.38] |
| Alford & Derzon (2012) | 41 | – | 3/4 | 1.27 [1.16, 1.40] |
| Bonell et al. (2016) | 3 | 3,201 | 3/3 | 1.04 [0.91, 1.18] |
| Park-Higgerson et al. (2008)^b^ | 7 | 6,790 | 3/6 | 1.04 [0.70, 1.55] |
| Durlak et al. (2011) | 112 | – | 2/2 | 1.49 [1.34, 1.69] |
| Barnes et al. (2014) | 19 | 29,049 | 2/3 | 1.39 [n.s.] |
| Grove et al. (2008) | 16 | 4,957 | 1/4 | 1.31 [1.06, 1.60] |
| Beelmann & Lösel (2021)^b^ | 5 | – | 0/1 | 1.18 [n.s.] |
| Wilson & Lipsey (2007) | 77 | – | 0/2 | 1.46 [*p* < 0.05] |
| **Psychosocial Interventions: (Cyber)bullying** | | | | |
| Jiménez-Barbero et al. (2016)^b^ | 14 | 30,934 | 4/6 | 1.24 [1.12, 1.36] |
| Chen et al. (2021) | 16 | 46,361 | 3/6 | 3.19 [1.54, 6.61] |
| Gaffney, Farrington et al. (2019) | 18 | 34,826 | 3/6 | 1.23 [1.04, 1.47] |
| Gaffney, Ttofi et al. (2019) | 81 | – | 2/5 | 1.31 [1.24, 1.39] |
| Ng et al. (2022); cyberbullying^b^ | 5 | 6,366 | 3/6 | 1.34 [1.06, 1.69] |
| Ng et al. (2022); bullying^b^ | 9 | 4,033 | 3/6 | 1.72 [1.31, 2.22] |
| Ferguson et al. (2007) | 26 | 28,185 | 2/6 | 1.39 [1.08, 1.73] |
| Merrell et al. (2008) | 8 | 11,942 | 1/2 | 1.08 [n.s.] |
| **Psychosocial Interventions: Sexual Violence** | | | | |
| Fellmeth et al. (2015)^b^ | 11 | 6,171 | 4/6 | 1.23 [0.95, 1.59] |
| Lee & Wong (2022) | 17 | 18,946 | 3/6 | 1.33 [1.11, 1.59] |
| De La Rue et al. (2017) | 4 | – | 2/3 | 1.22 [1.02, 1.46] |
| Wright et al. (2018) | 5 | 406 | 2/4 | 1.06 [0.74, 1.52] |
| Eggers del Campo & Steinert (2022)^b^ | 14 | 24,079 | 1/4 | 1.20 [1.06, 1.36] |
| **Physical Activity** | | | | |
| Harwood et al. (2017) | 8 | 459 | 2/4 | 2.66 [2.48, 2.86] |
| Spruit et al. (2016) | 6 | – | 0/1 | 1.71 [n.s.] |
| **Community-based/legal** | | | | |
| Piza et al. (2019) | 29 | – | 3/5 | 1.05 [0.95, 1.16] |
| Sadatsafavi et al. (2022); combined | 10 | 76,818^c^ | 3/6 | 1.12 [1.06, 1.16] |
| Sadatsafavi et al. (2022); mowing | 3 | 21,526^c^ | 3/6 | 1.12 [0.95, 1.31] |
| Sadatsafavi et al. (2022); greening | 5 | 54,044^c^ | 3/6 | 1.12 [1.08, 1.16] |
| Sadatsafavi et al. (2022); gardening | 2 | 1,248^c^ | 3/6 | 1.10 [1.06, 1.16] |
| Telep et al. (2014) | 4 |  | 3/5 | 1.58 [1.24, 2.02] |
| Wagenaar et al. (2010) | 10 | – | 1/6 | 1.08 [1.04, 1.13] |
| Makarios & Pratt (2012); combined | 29 | – | 0/1 | 1.70 [*p* < 0.05] |
| Makarios & Pratt (2012); gun buy-backs | – | – | 0/1 | 1.04 [n.s.] |
| Makarios & Pratt (2012); gun laws | – | – | 0/1 | 1.38 [*p* < 0.05] |
| Makarios & Pratt (2012); law enforcement | – | – | 0/1 | 2.37 [*p* < 0.05] |

Note. *k* = number of studies included in a meta-analysis; *n* = number of participants included in a meta-analysis; OR = odds ratio; CI = confidence interval; n.s. = not significant. *p* < .05 = statistically significant with a confidence level smaller than .05. Quality Score ranges from 0 (low quality) to 6 (high quality), and reports: no. positive quality items/no. quality items reported. “Mowing” interventions: trash/debris removal, mowing vegetation regularly. “Greening” interventions: trash/debris removal, grading land, planting new grass/trees, installing fences, maintaining lots. “Gardening” interventions: grading soil, planting turfgrass, mowing, various landscaping.

^a^ The analyses were re-ran without one clear outlier (OR > 230), which skewed the overall effect size due to the use of a random effects model. When the outlier is included in the analysis, the overall effect is: 2.79 [1.15, 6.77].

^b^ Meta-analysis based entirely on randomized controlled trials (RCTs).

^c^ Number of lots observed.

**Supplementary Table 2a**

*Characteristics of all Included Meta-Analyses*

| **Study** | **Intervention** | **Population** | **Outcome** |
| --- | --- | --- | --- |
| Alford & Derzon (2012) | School-based interventions to reduce violence and antisocial behaviour | School-aged children and youths | Physical aggression, antisocial behaviour, aggressive/disruptive behaviour, and delinquent behaviour |
| Barnes et al. (2014) | School-based cognitive-behavioural interventions | School-aged children and youths | Aggression |
| Beelmann & Lösel (2021) | Social skills training programmes | Children and youths (0-18 years) | Antisocial behaviour (including aggression, delinquency, and violence) |
| Bonell et al. (2016) | Positive youth development interventions delivered outside of school | Children and youths (11-18 years) | Physical violence |
| Chen et al. (2021) | School and home-based anti-bullying interventions with parental involvement | School-aged children | Bullying |
| De La Rue et al. (2017) | School-based interventions aimed at preventing teen dating violence | Children and youths (11-18 years) | Dating violence perpetration |
| Durlak et al. (2011) | School-based social and emotional learning interventions | Children and youths (5-18 years) | Conduct problems (including aggression, bullying, and disruptive behaviour) |
| Eggers del Campo & Steinert (2022) | Female economic empowerment interventions | Women (and men) from low/middle income countries | Physical violence |
| Fellmeth et al. (2015) | Emotional and skills-based interventions aimed at relationship violence | Adolescents and young adults (12-25 years) | Episodes of dating and relationship violence |
| Ferguson et al. (2007) | School-based anti-bullying interventions | School-aged children and youths | Bullying and other serious violent behaviour |
| Gaffney, Farrington et al. (2019) | Anti-cyberbullying interventions | School-aged children and youths | Cyberbullying |
| Gaffney, Ttofi et al. (2019) | School-based anti-bullying interventions | School-aged children and youths | School-bullying |
| Grove et al. (2008) | Prevention programmes of primary behavioural problems associated with ODD and CD | Children and youths (up until 19 years) | Aggression |
| Harwood et al. (2017) | Martial arts | Children and youths (up until 18 years) | Externalizing behaviour (including aggression, anger, violence, and hostility) |
| Jiménez-Barbero et al. (2016) | School-based anti-bullying interventions | Children and youths (7-16 years) | Bullying or school-violence frequency |
| Langford et al. (2014) | Interventions based on the WHO Health Promoting Schools (HPS) framework | Children and youths (4-18 years) | Violence and bullying |
| Lee & Wong (2022) | Dating violence prevention programmes | Adolescents (< 18 years) | Dating violence perpetration |
| Makarios & Pratt (2012) | Policies aimed at reducing gun violence | General population | Gun violence |
| Merrell et al. (2008) | School-based anti-bullying interventions | School-aged children and youths | Bullying |
| Moy & Hazen (2018) | Second Step interventions (i.e., social emotional learning) | School-aged children | Antisocial behaviour |
| Ng et al. (2022) | School-based anti-bullying interventions | Children and youths (10-19 years) | Bullying and cyberbullying |
| Park-Higgerson et al. (2008) | School-based violence prevention interventions | School-aged children and youths | Externalizing, aggressive, or violent behaviour |
| Piza et al. (2019) | CCTV surveillance | General population | Violent crime |
| Sadatsafavi et al. (2022) | City-wide vacant lot remediation programmes | General population | Firearm violence |
| Spencer et al. (2021) | Online-based programmes aimed at reducing intimate partner violence | General population | Intimate partner violence perpetration |
| Spruit et al. (2016) | Physical activity interventions | Children and youths (10-21 years old) | Externalizing problems |
| Telep et al. (2014) | Formal social control interventions (e.g., policy changes) | General population | Violent crime |
| Wagenaar et al. (2010) | Alcohol tax and price policies | General population | General and sexual violence |
| Wilson & Lipsey (2007) | School-based interventions aimed at reducing aggressive and disruptive behaviour | School-aged children | Aggressive and disruptive behaviour |
| Wright et al. (2018) | Male-targeted sexual assault prevention programmes | Adult males (> 18 years) | Self-reported perpetrated sexual aggression |

**Supplementary Table 2b**

*Designs Used in Main Meta-analyses*

| **Study** | **Research Designs** |
| --- | --- |
| Bonell et al. (2016) | RCTs and prospective studies with non-random matched control groups. |
| Durlak et al. (2011) | Various quasi-experimental designs with a control group. |
| Eggers del Campo & Steinert (2022) | RCTs. |
| Gaffney, Farrington et al. (2019) | Experimental studies with one group receiving the intervention and another (control group) not receiving the intervention. |
| Gaffney, Ttofi et al. (2019) | Experimental or quasi-experimental design, with one group receiving the intervention and another (control group) not receiving the intervention. |
| Lee & Wong (2022) | Various quasi-experimental designs with a control group or a repeated measures design. |
| Harwood et al. (2017) | Various quasi-experimental designs with a control group. |
| Makarios & Pratt (2012) | Various. |
| Moy & Hazen (2018) | One of (a) experimental or quasi-experimental design that compared students exposed to the Second Step program with one or more comparison conditions on at least one qualifying outcome variable; or (b) a repeated measures design in which measures of at least one qualifying outcome variable were taken before and after intervention on the same participants. |
| Piza et al. (2019) | Before-and-after measures of crime in treatment and comparable control areas. |
| Sadatsafavi et al. (2022) | Before-and-after studies with control group. |
| Spencer et al. (2021) | Various quasi-experimental designs with a control group. |
| Spruit et al. (2016) | Experimental (i.e., treatment group was compared to a comparison group of juveniles who did not participate in a physical activity intervention). |
| Telep et al. (2014) | Randomized experiments or quasi-experiments with a comparison group that did not receive the intervention or change in conditions, as well as quasi-experiments that adjusted for secular trends (e.g., citywide crime rates). |
| Wagenaar et al. (2010) | Ecological designs. |
| Wright et al. (2018) | Various. |

**Supplementary Table 3**

*Quality Assessment of all Included Reviews*

| **Study** | **AMSTAR** | **Number of cases > 1000** | **Prediction interval excludes null value** | **Heterogeneity** | **Small study effects** | **Statistical excess** | **Total score (max = 6)** |
| --- | --- | --- | --- | --- | --- | --- | --- |
| Alford & Derzon (2012) | Low | – | Yes | Low | No | – | 3 |
| Barnes et al. (2014) | Medium | Yes | – | Low | – | – | 2 |
| Beelmann & Lösel (2021) | Medium | – | – | – | – | – | 0 |
| **Bonell et al. (2016)** | High | Yes | – | Low | – | – | 3 |
| Chen et al. (2021) | High | Yes | No | High | No | Yes | 3 |
| De La Rue et al. (2017) | High | – | No | Low | – | – | 2 |
| **Durlak et al. (2011)** | High | – | – | Low | – | – | 2 |
| **Eggers del Campo & Steinert (2022)** | Medium | Yes | – | High | – | Yes | 1 |
| Fellmeth et al. (2015) | High | Yes | No | Low | No | Yes | 4 |
| Ferguson et al. (2007) | Medium | Yes | No | High | No | Yes | 2 |
| **Gaffney, Farrington et al. (2019)** | High | Yes | No | High | No | Yes | 3 |
| **Gaffney, Ttofi et al. (2019)** | High | – | No | High | No | Yes | 2 |
| Grove et al. (2008) | Medium | Yes | – | High | – | Yes | 1 |
| **Harwood et al. (2017)** | Medium | No | – | Low | – | No | 2 |
| Jiménez-Barbero et al. (2016) | High | Yes | No | High | No | No | 4 |
| Langford et al. (2014) | High | Yes | No | High | No | No | 4 |
| **Lee & Wong (2022)** | Medium | Yes | No | High | No | No | 3 |
| **Makarios & Pratt (2012)** | Medium | – | – | – | – | – | 0 |
| Merrell et al. (2008) | Medium | Yes | – | – | – | – | 1 |
| **Moy & Hazen (2018)** | High | Yes | No | High | No | No | 4 |
| Ng et al. (2022) | High | Yes | No | High | No | Yes | 3 |
| Park-Higgerson et al. (2008) | Medium | Yes | No | High | No | No | 3 |
| **Piza et al. (2019)** | High | – | No | High | No | No | 3 |
| **Sadatsafavi et al. (2022)** | Medium | Yes | No | High | No | No | 3 |
| **Spencer et al. (2021)** | Medium | Yes | No | Low | No | No | 4 |
| **Spruit et al. (2016)** | Medium | – | – | – | – | – | 0 |
| **Telep et al. (2014)** | High | – | No | Low | No | Yes | 3 |
| **Wagenaar et al. (2010)** | Medium | Yes | No | High | Yes | Yes | 1 |
| Wilson & Lipsey (2007) | Medium | – | – | High | – | – | 0 |
| **Wrigth et al. (2018)** | High | No | – | Low | – | Yes | 2 |

*Note*. Studies in BOLD are included in the main analyses. High AMSTAR rating = 1; number of cases > 1000 = 1; prediction interval excluding null value = 1; low heterogeneity (I^2^ < 50%) = 1; no evidence to suggest small study effects = 1; no indication of statistical excess = 1; missing information (–) = 0.

Studies in bold are reviews used as part of the main findings. The total score (max = 6) is potentially underestimated for studies with missing data on quality assessment items.

# References

Alford, A. A., & Derzon, J. (2012). Meta-analysis and systematic review of the effectiveness of school-based programs to reduce multiple violent and antisocial behavioral outcomes. In *Handbook of School Violence and School Safety*. Routledge. <https://doi.org/10.4324/9780203841372.ch44>

Barnes, T. N., Smith, S. W., & Miller, M. D. (2014). School-based cognitive-behavioral interventions in the treatment of aggression in the United States: A meta-analysis. *Aggression and Violent Behavior*, *19*(4), 311–321. <https://doi.org/10.1016/j.avb.2014.04.013>

Beelmann, A., & Lösel, F. (2021). A comprehensive meta-analysis of randomized evaluations of the effect of child social skills training on antisocial development. *Journal of Developmental and Life-Course Criminology*, *7*(1), 41–65. <https://doi.org/10.1007/s40865-020-00142-8>

Bonell, C., Dickson, K., Hinds, K., Melendez-Torres, G., Stansfield, C., Fletcher, A., Thomas, J., Lester, K., Oliver, E., Murphy, S., & Campbell, R. (2016). The effects of Positive Youth Development interventions on substance use, violence and inequalities: Systematic review of theories of change, processes and outcomes. *Public Health Research*, *4*(5), 1–218. <https://doi.org/20160520141157162>

Chen, Q., Zhu, Y., & Chui, W. H. (2021). A meta-analysis on effects of parenting programs on bullying prevention. *Trauma, Violence, & Abuse*, *22*(5), 1209–1220. <https://doi.org/10.1177/1524838020915619>

De La Rue, L., Polanin, J. R., Espelage, D. L., & Pigott, T. D. (2017). A meta-analysis of school-based interventions aimed to prevent or reduce violence in teen dating relationships. *Review of Educational Research*, *87*(1), 7–34. <https://doi.org/10.3102/0034654316632061>

Durlak, J. A., Weissberg, R. P., Dymnicki, A. B., Taylor, R. D., & Schellinger, K. B. (2011). The impact of enhancing students’ social and emotional learning: A meta-analysis of school-based universal interventions: social and emotional learning. *Child Development*, *82*(1), 405–432. <https://doi.org/10.1111/j.1467-8624.2010.01564.x>

Eggers del Campo, I., & Steinert, J. I. (2022). The effect of female economic empowerment interventions on the risk of intimate partner violence: A systematic review and meta-analysis. *Trauma, Violence, & Abuse*, *23*(3), 810–826. <https://doi.org/10.1177/1524838020976088>

Fellmeth, G., Heffernan, C., Nurse, J., Habibula, S., & Sethi, D. (2015). Educational and skills-based interventions to prevent relationship violence in young people. *Research on Social Work Practice*, *25*(1), 90–102. <https://doi.org/10.1177/1049731514533392>

Ferguson, C. J., Miguel, C. S., Kilburn, J. C., & Sanchez, P. (2007). The effectiveness of school-based anti-bullying programs: A meta-analytic review. *Criminal Justice Review*, *32*(4), 401–414. <https://doi.org/10.1177/0734016807311712>

Gaffney, H., Farrington, D. P., Espelage, D. L., & Ttofi, M. M. (2019). Are cyberbullying intervention and prevention programs effective? A systematic and meta-analytical review. *Aggression and Violent Behavior*, *45*, 134–153. <https://doi.org/10.1016/j.avb.2018.07.002>

Gaffney, H., Ttofi, M. M., & Farrington, D. P. (2019). Evaluating the effectiveness of school-bullying prevention programs: An updated meta-analytical review. *Aggression and Violent Behavior*, *45*, 111–133. <https://doi.org/10.1016/j.avb.2018.07.001>

Grove, A. B., Evans, S. W., Pastor, D. A., & Mack, S. D. (2008). A meta-analytic examination of follow-up studies of programs designed to prevent the primary symptoms of oppositional defiant and conduct disorders. *Aggression and Violent Behavior*, *13*(3), 169–184. <https://doi.org/10.1016/j.avb.2008.03.001>

Harwood, A., Lavidor, M., & Rassovsky, Y. (2017). Reducing aggression with martial arts: A meta-analysis of child and youth studies. *Aggression and Violent Behavior*, *34*, 96–101. <https://doi.org/10.1016/j.avb.2017.03.001>

Jiménez-Barbero, J. A., Ruiz-Hernández, J. A., Llor-Zaragoza, L., Pérez-García, M., & Llor-Esteban, B. (2016). Effectiveness of anti-bullying school programs: A meta-analysis. *Children and Youth Services Review*, *61*, 165–175. <https://doi.org/10.1016/j.childyouth.2015.12.015>

Langford, R., Bonell, C. P., Jones, H. E., Pouliou, T., Murphy, S. M., Waters, E., Komro, K. A., Gibbs, L. F., Magnus, D., & Campbell, R. (2014). The WHO Health Promoting School framework for improving the health and well-being of students and their academic achievement. *Cochrane Database of Systematic Reviews*. <https://doi.org/10.1002/14651858.CD008958.pub2>

Lee, C., & Wong, J. S. (2022). Examining the effects of teen dating violence prevention programs: A systematic review and meta-analysis. *Journal of Experimental Criminology*, *18*(1), 1–40. <https://doi.org/10.1007/s11292-020-09442-x>

Makarios, M. D., & Pratt, T. C. (2012). The effectiveness of policies and programs that attempt to reduce firearm violence: A meta-analysis. *Crime & Delinquency*, *58*(2), 222–244. <https://doi.org/10.1177/0011128708321321>

Merrell, K. W., Gueldner, B. A., Ross, S. W., & Isava, D. M. (2008). How effective are school bullying intervention programs? A meta-analysis of intervention research. *School Psychology Quarterly*, *23*(1), 26–42. <https://doi.org/10.1037/1045-3830.23.1.26>

Moy, G. E., & Hazen, A. (2018). A systematic review of the Second Step program. *Journal of School Psychology*, *71*, 18–41. <https://doi.org/10.1016/j.jsp.2018.10.006>

Ng, E. D., Chua, J. Y. X., & Shorey, S. (2022). The effectiveness of educational interventions on traditional bullying and cyberbullying among adolescents: A systematic review and meta-analysis. *Trauma, Violence, & Abuse*, *23*(1), 132–151. <https://doi.org/10.1177/1524838020933867>

Park-Higgerson, H.-K., Perumean-Chaney, S. E., Bartolucci, A. A., Grimley, D. M., & Singh, K. P. (2008). The evaluation of school-based violence prevention programs: A meta-analysis. *Journal of School Health*, *78*(9), 465–479. <https://doi.org/10.1111/j.1746-1561.2008.00332.x>

Piza, E. L., Welsh, B. C., Farrington, D. P., & Thomas, A. L. (2019). CCTV surveillance for crime prevention: A 40‐year systematic review with meta‐analysis. *Criminology & Public Policy*, *18*(1), 135–159. <https://doi.org/10.1111/1745-9133.12419>

Sadatsafavi, H., Sachs, N. A., Shepley, M. M., Kondo, M. C., & Barankevich, R. A. (2022). Vacant lot remediation and firearm violence – A meta-analysis and benefit-to-cost evaluation. *Landscape and Urban Planning*, *218*, 104281. <https://doi.org/10.1016/j.landurbplan.2021.104281>

Spencer, C. M., Stith, S. M., & King, E. L. (2021). Preventing maltreatment at home: A meta-analysis examining outcomes from online programs. *Research on Social Work Practice*, *31*(2), 138–146. <https://doi.org/10.1177/1049731520969978>

Spruit, A., Assink, M., van Vugt, E., van der Put, C., & Stams, G. J. (2016). The effects of physical activity interventions on psychosocial outcomes in adolescents: A meta-analytic review. *Clinical Psychology Review*, *45*, 56–71. <https://doi.org/10.1016/j.cpr.2016.03.006>

Telep, C. W., Weisburd, D., Gill, C. E., Vitter, Z., & Teichman, D. (2014). Displacement of crime and diffusion of crime control benefits in large-scale geographic areas: A systematic review. *Journal of Experimental Criminology*, *10*(4), 515–548. <https://doi.org/10.1007/s11292-014-9208-5>

Wagenaar, A. C., Tobler, A. L., & Komro, K. A. (2010). Effects of alcohol tax and price policies on morbidity and mortality: A systematic review. *American Journal of Public Health*, *100*(11), 2270–2278. <https://doi.org/10.2105/AJPH.2009.186007>

Wilson, S. J., & Lipsey, M. W. (2007). School-based interventions for aggressive and disruptive behavior. *American Journal of Preventive Medicine*, *33*(2), S130–S143. <https://doi.org/10.1016/j.amepre.2007.04.011>

Wright, L. A., Zounlome, N. O. O., & Whiston, S. C. (2018). The effectiveness of male-targeted sexual assault prevention programs: A meta-analysis. *Trauma, Violence, & Abuse*, *21*(5), 859–869. <https://doi.org/10.1177/1524838018801330>
